# Retraction: Does China Pakistan Economic Corridor become an avenue to achieve sustainable development goal no. 2 (food security) in Pakistan: Under the condition of COVID-19?

**DOI:** 10.1371/journal.pone.0284214

**Published:** 2023-04-04

**Authors:** The PLOS ONE Editors

Following the publication of this article and Correction [1, 2], concerns were raised regarding similarity to a previously published article with no shared authors [3], and the consistency and validity of survey questions based on files provided during the review process.

Similarities between the articles [1, 3] include potential paraphrasing throughout the results section, and similar figures, including Fig 4 of [1] which appears to have irregularities in the size, format and alignment of text and numbers, and which appears similar to Fig 3 of [3].

The corresponding author stated that there is no text overlap, and similarities may have occurred due to the use of similar methodology and analysis of some of the same variables. They also said that the figures are not the same in the two articles, and that similar figure layout could be due to the use of similar methodology. The editors remain concerned about the high similarities between these articles [1, 3].

In relation to the consistency and validity of survey questions, the corresponding author confirmed that they did not provide the same questionnaire to all stakeholders, and indicated that this was due to differences between different professions. They did not comment on the concerns regarding validity of the survey questions. The editors remain concerned about the reliability of survey results due to these concerns.

In light of the unresolved concerns, the *PLOS ONE* Editors retract this article.

All authors agreed with the retraction and apologized for the issues with the published article.
